# Aretaeus of Cappadocia and Pulmonary Tuberculosis

**DOI:** 10.4274/balkanmedj.2016.1847

**Published:** 2017-09-29

**Authors:** Konstantinos Laios, George Androutsos, Marilita M. Moschos

**Affiliations:** 1 Department of Ophthalmology, Medical School, National and Kapodistrian University of Athens, Athens, Greece; 2 Biomedical Research Foundation, Academy of Athens, Athens, Greece

To the Editor,

Aretaeus of Cappadocia is a controversial figure in the history of ancient Greek medicine. Whether he was a victim of plagiarism or a plagiarist initiated a heated debate about his dating, which fluctuated from 1^st^ BC c. to 4^th^ AD c., although most scholars prefer 2^nd^ AD c. (1). Nevertheless, no one denied that he was a physician who formed the most accurate descriptions of diseases in antiquity (2). Such an example is his to the point analysis about phthisis, which, according to modern medicine, can be identified as pulmonary tuberculosis in Chapter VIII of his First Book on Chronic Diseases (3).

He started defining the cause of the disease by stating that phthisis was the condition where a pulmonary ulcer or an abscess was present, while the patient had a lot of cough and expectoration, while sometimes haemoptysis was also a symptom. He differentiated the disease from the presence of purulence in the pleura, a fact that pointed to his ability for accurate clinical description.

He underlined the two major symptoms of the disease. One was fever, which could be continuous and high or latent. If the patient remained afebrile during the day only, the physician believed that the fever never actually stopped but could not be detected due to the sweat and the coldness of the environment during the day.

The other symptom, to which he devoted the majority of the chapter, was emaciation. He wrote about the great loss of weight and the weakness of the body. It is surprising that he spoke about the emaciation of each part of the human body, beginning from the head and reaching to the limbs, giving an elaborate description, which was unparalleled in ancient Greek medical literature, underlining that one could see almost all the bones of the body under the skin and especially those of the thorax. Very important were his observations of patients’ clubbing fingers, a unique description among ancient Greek medical texts, believing that it was an outcome of the emaciation of the limbs.

He finished the chapter by pointing out that mostly young people were affected, those who could avoid death, distinguishing them from older people who rarely presented the disease but seldom survived.

Although all major ancient Greek physicians wrote about phthisis, no other overall presentation of the disease has been preserved (4). Furthermore, no one managed as Aretaeus of Cappadocia to highlight synoptically its most important pathological signs. Therefore, he gave us its most accurate and detailed semiology, which was useful for each ancient physician in his daily work. The other physicians did not summarize the symptoms of the disease but pointed out only a few of them. In addition, the most extensive presentation of the disease in ancient times, that of Caelius Aurelianus (5^th^ AD c.), which was a Latin translation of Soranus of Ephesus (1^st^/2^nd^ AD c.) work (5), was devoted to the treatment of the disease, while the cause and the signs were limited to only few paragraphs, being inferior in comparison to that of Aretaeus of Cappadocia.
